# Complete response of radioresistant brain metastases from non-small cell lung cancer with temozolomide: A case report and literature review

**DOI:** 10.1097/MD.0000000000023592

**Published:** 2020-12-18

**Authors:** Yi Yang, Yu Pu, Nan Dai, Dong Wang, Mingfang Xu

**Affiliations:** Cancer Center, Daping Hospital, Army Medical University, Chongqing 400042, P.R. China.

**Keywords:** brain metastases, non-small cell lung cancer, temozolomide

## Abstract

**Rationale:**

Non-small cell lung cancer (NSCLC) patients with brain metastases (BMs) have been found as subjects of poor prognosis. Whole-brain radiotherapy (WBRT), surgery, and stereotactic radiosurgery, epidermal growth factor receptor tyrosine kinase inhibitor (EGFR-TKI), or some combinations are the most commonly employed strategies for the treatment of treatments BMs. However, some patients are resistant to all these treatments.

**Patient Concerns:**

We present an NSCLC patient with progression of BMs after treatment with WBRT and EGFR-TKIs. The patient was diagnosed with multiple metastases on July 9, 2014, and treated with docetaxel plus cisplatin chemotherapy followed with gefitinib as the maintenance therapy. The patient showed recurrence of BMs after 8-months of chemotherapy. WBRT with 30 Gy was administrated in 10 fractions. Tumor progression of the brain was diagnosed with an magnetic resonance imaging scan after 2-months of WBRT.

**Diagnoses:**

The patient was diagnosed as pulmonary adenocarcinoma with diffuse metastases in both lungs and multiple metastases in bone and brain. Progression of BMs was confirmed through magnetic resonance imaging.

**Interventions:**

This patient was administered temozolomide (150 mg/m^2^/d for 5 days every 28-day cycle). As a whole, 6 cycles were performed after the progression of BMs from August 2015.

**Outcomes:**

The patient got complete brain remission and lived without discomfort. The intracranial lesion did not progress until the progression of the lung lesion and led to death on February 20, 2019. The intracranial progression-free survival was 42 months, whereas the overall survival was 55 months.

**Lessons:**

For patients with NSCLC and BMs, temozolomide can be used as a treatment option, especially in patients with EGFR-TKIs resistance or without driver mutations.

## 1 Introduction

Lung cancer is considered one of the most common causes of cancer-related deaths worldwide^[1]^ and accounts for approximately half of all brain metastases (BMs).^[2]^ Furthermore, the incidence of BMs has significantly increased over the last years.^[3]^ In particular, 44% to 60% of lung adenocarcinoma patients with the driver gene mutation developed BMs during their disease period.^[4]^ Once BMs occur in non-small cell lung cancer (NSCLC) patients, the observed median overall survival (OS) is approximately 6 months.^[5,6]^

Treatment of patients with BMs depends upon the number of metastases, general condition of patients, and the driver mutations. Surgical resection or stereotactic radiosurgery (SRS) can be the treatment of choice for single BMs. After surgical resection, postoperative whole-brain radiotherapy (WBRT), or SRS is generally recommended.^[7]^ Patients having a good prognosis and better survival factor, that is, driver mutations are recommended to undergo WBRT. The most frequent schedule of WBRT includes 30 Gy in 10 fractions or 20 Gy in 5 fractions, where both the modalities provide similar outcomes. The QUARTZ trial data^[8]^ showed that patients with less than 70 Karnofsky score did not get benefit from WBRT treatment. Next-generation tyrosine kinase inhibitors (TKIs) are capable of restoring the control of disease and cause a delay in cranial radiation therapy in patients with driver mutations (eg, epidermal growth factor receptor [EGFR], anaplastic lymphoma kinase [ALK]), and asymptomatic BMs. However, some patients are resistant to all of the above treatments. There are only a few treatment options available for BMs patients who are resistant to radiotherapy and TKIs.

Temozolomide (TMZ) is an active drug molecule and is used for the treatment of patients having gliomas and O6-methylgaunine-DNA-methyltransferase (MGMT) promoter methylation is an independent prognostic factor.^[9]^ Besides, TMZ combination with olaparib has also achieved good results in the treatment of small cell lung cancer patients. TMZ has a high affinity to cross the blood-brain barrier (BBB), thus leading to localization of its therapeutic concentration in the brain.^[10,11]^ However, its activity remains controversial in NSCLC patients with BMs in a series of phase II or phase III trials.^[12–14]^ Routine use of chemotherapy alone for BMs is not recommended as it has not been efficient in improving the OS.^[15]^

Here, we report an NSCLC patient with the progression of BMs after treatment with WBRT and epidermal growth factor receptor tyrosine kinase inhibitors (EGFR-TKIs). The patient received the treatment of TMZ at a dose of 150 mg/m^2^/d for 5 days every 28-day cycle with a total of 6 cycles and got complete remission in the brain without obvious side effects. The intracranial progression-free survival (PFS) was 42 months, and OS was 55 months. We believe that TMZ represents a promising treatment strategy for patients with radio-resistance in BMs from NSCLC. The patient provided written informed consent for publication of the case details. The approval of this study was granted by the ethics committee of Daping Hospital (Chongqing, China).

## 2 Case presentation

In the Cancer Center of our hospital, a 46-years old female patient was admitted due to an 8-months history of irritable cough. The patient had pain in the back for 1 month. At the time of admission, the performance status was grade 1 following the Eastern Cooperative Oncology Group scale. Diagnosis of chronic non-atrophic gastritis had been included in the medical history of the patient in 2001 with no previous drinking or smoking history.

The patient underwent computed tomography (CT)-guided percutaneous intrathoracic lung biopsy in July 2014 and was diagnosed as pulmonary adenocarcinoma with EGFR exon 19 deletion mutation. Analysis based on genome sequencing revealed MGMT gene mutation in exon1, exon4, and exon5 of lung cancer tissue (Table 1). Tumor assessments at baseline included the chest and abdomen CT scan (contrast-enhanced). Screening of bone metastases was performed with emission computed tomography (ECT) while the diagnosis was authenticated by CT and contrast-enhanced magnetic resonance imaging (MRI) scan of the brain. Investigator-assessed Response Evaluation Criteria in Solid Tumors version 1.1 were employed for the determination of objective responses. CT images showed that there were diffuse military metastases in both lungs. ECT images demonstrated multiple bone metastases, and MRI showed multiple BMs. The cancer was classified as stage IV (T4N1M1b).

**Table 1:** O6-methylgaunine-DNA-methyltransferase gene mutation status based on whole genomic sequencing.

			Mutation frequency	
Gene	Exon	Amino acid variation	Cell free DNA	Lung tumor DNA
MGMT	exon1	p.P5S	0.76%	1.35%
MGMT	exon4	p.S124L	–	5.08%
MGMT	exon4	p.E141K	–	2.16%
MGMT	exon5	p.Y189C	–	2.43%
MGMT	exon5	p.E203∗	–	4.21%
MGMT	exon5	p.S216L	–	7.41%
MGMT	exon5	p.A226V	–	4.35%
MGMT	exon5	p.P233S	0.29%	1.49%
MGMT	exon5	p.P233L	–	4.2%
MGMT	exon5	p.P234H	–	4.97%
MGMT	exon5	p.R237∗	–	2.23%

**Figure 1:**
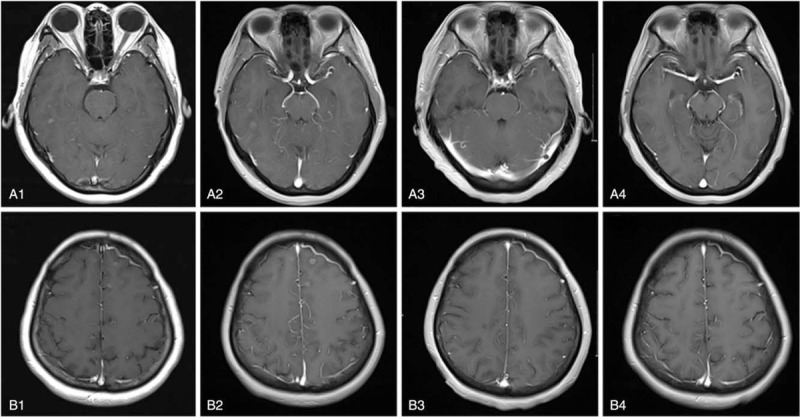
MRI findings in the patient presented with brain metastases. A and B represent a different site. 1, 2, 3, and 4 represent represents different times. A1 and B1. Enhanced, Coronal MRI images demonstrated masses in the cerebral hemisphere on May 12, 2015, and the patient's intracranial lesion progressed. A2 and B2. Enhanced, Coronal MRI images demonstrated masses increase in the size in the cerebral hemisphere on July 29, 2015. The patient's intracranial lesion progressed again. A3 and B3. Enhanced, Coronal MRI images demonstrated no mass in the cerebral t hemisphere on October 20, 2015. The patient's brain metastases disappeared completely. A4 and B4. Enhanced, Coronal MRI images demonstrated no recurrence of brain metastases on November 13, 2018 (39 mo after TMZ). MRI = magnetic resonance imaging, TMZ = temozolomide.

The patient received 6 cycles of docetaxel plus cisplatin chemotherapy regimen, including docetaxel (75 mg/m^2^; days 1; intravenous), cisplatin (75 mg/m^2^; days 1; intravenous), followed by a daily dosage of gefitinib (250 mg) as maintenance therapy. The only side effects of chemotherapy were alopecia (III°), and there was no obvious side effect in gefitinib treatment. CT scan showed that the pulmonary lesions were reduced. ECT and MRI respectively showed stable bones and brain after chemotherapy. After 8 months of chemotherapy, MRI revealed that the central nervous system (CNS) lesions (Fig. 1A1 and B1) increased in size as well as the number of tumors. Objective efficacy evaluation indicated the progression of BMs. CT revealed the partial response of lungs, while ECT revealed stable bone.

Then, the patient received WBRT with 30 Gy dosing administered in 10 fractions of 3 Gy. MRI revealed an increase in the size of CNS lesions as well in the number of tumors (Fig. 1A2 and B2) 2-months after WBRT. Objective efficacy evaluation indicated the progression of BMs, and new lesions in the brain suggested that it was not pseudoprogression. She complained of dizziness and headache.

Thereafter, the patient was administrated TMZ (150 mg/m^2^/d for 5 days every 28-day cycle, with a total of 6 cycles) from August 2015. Because of stability in extracranial lesions, the patient continued gefitinib treatment. If needed, oral dosing of 8 mg of Ondansetron was also provided before TMZ. The major adverse reactions were nausea and Vomiting (grade I, according to the National Cancer Institute Common Terminology Criteria for Adverse Events, version 3.0). After 2-cycles, follow up scans were obtained for assessing responses. MRI imaging revealed the disappearance of BMs (Fig. 1A3 and B3). Thereafter, the patient had no symptoms associated with BMs until death. Since then, the patient had no symptoms related to BMs. After 39-months of TMZ, the intracranial lesions did not persist on MRI imaging (Fig. 1A4 and B4). The intracranial lesion did not progress until the progression of the extracranial lesion and led to death in February 2019. The intracranial PFS was 42 months, while OS was 55 months.

## 3 Discussion

BMs are frequent causes of death in people with lung cancer and have been classified as the most frequent type of malignancy in the brain. Generally, the treatment of patients with BMs includes surgery, WBRT, SRS, EGFR tyrosine kinase inhibitors, or combinations of these treatment modalities. Currently, WBRT is considered a standard initial treatment for multiple BMs regardless of histology. Unfortunately, WBRT is associated with responders’ late-onset progressive cognitive impairment, thus, this in turn limits its clinical applications.^[16]^ WBRT is considered safe if it avoids affecting the hippocampus,^[17]^ however, it is not recommended for routine care as it is still in the trial phase. A recent research study reveals that SRS can be considered as an effective and safe standard alternative treatment strategy to WBRT. According to a meta-analysis,^[18]^ when compared with surgery, there was no conclusive proof regarding the SRS safety and effectiveness in terms of OS, PFS, adverse events, and quality of life in people with single BMs. After complete resection of 1 to 3 BMs, SRS of the operating cavity significantly reduced the local reappearance as compared to the observation alone.^[19]^

The high incidence rate of BMs in patients having lung adenocarcinoma with a driver gene mutation is an unavoidable health issue. The use of CNS-penetrant next-generation TKIs such as alectinib and osimertinib in patients with actionable oncogenic drivers (such as ALK and EGFR) can restore the control of brain diseases, thus, significantly delay the radiotherapy of the brain.^[20,21]^ EGFR and TKIs combined with concomitant WBRT can control intracranial lesions of lung cancer metastasis and significantly improve patients’ survival.^[22]^ Furthermore, bevacizumab combined with EGFR-TKIs prolonged median progress-free survival (14.0 vs 8.2 months; *P* < .001) and median OS (14.4 vs 9.0 months; *P* < .001) in EGFR-mutant NSCLC patients with multiple BMs as compared to EGFR-TKIs alone. Hence, this treatment (bevacizumab-EGFR-TKIs combination) is considered an effective and safe alternative for these patients.^[23]^ However, some patients are resistant to all those treatments due to primary or acquired resistance.

Generally, there is no standard treatment for progressive BMs from lung cancer. In this study, we present a TMZ treated patient after the disease progression to CNS following radiation therapy and EGFR-TKIs. The BMs completely disappeared after the administration of TMZ, indicating that TMZ has shown activity against BMs from NSCLC. In the patient, gefitinib was effective against the lung primary lesions, but not against BMs due to low CNS-concentration of gefitinib and tumor heterogeneity, which is common in lung cancer. Chemotherapy remains controversial in BMs patients due to the BBB which prevents the permeation of chemotherapy agents into the brain.^[24]^ TMZ provides an effective concentration in the brain because of its ability to cross the BBB.^[11]^ The observed TMZ concentration in the cerebrospinal fluid is almost 20% of serum concentrations.^[10]^ But the TMZ treatment is ineffective for the treatment of NSCLC patients with BMs. The meta-analysis based on 14 eligible randomized controlled trials verified that the objective response rate was better with combined therapy of WBRT and TMZ than with WBRT alone (Relative Risk = 1.38, *P* < .00001) in NSCLC patients.^[25]^ On the other hand, a phase III trial^[26]^ suggested a poor outcome for NSCLC patients with BMs upon simultaneous treatment of TMZ and WBRT as compared to WBRT alone. At the same time, WBRT and TMZ resulted in a high probability for brain toxicity, and predominantly neurocognitive deficits.^[27]^

As a single agent, the TMZ efficacy is limited for NSCLC patients with BMs. Several phase II studies present the treatment by TMZ alone serving as salvage therapy for the patients having progressive lesions after WBRT revealed optimistic outcomes.^[28,29]^ On the other hand, in the phase II study TMZ alone was found to be inactive while treating the patients with BMs.^[14]^ However, our results reveal the effectiveness and safety of TMZ. Moreover, TMZ extended the PFS of the patient. We presume contradiction in results due to the MGMT gene mutation status.

MGMT promoter methylation is an independent favorable prognostic factor of gliomas.^[9]^ A meta-analysis analyzing 15 articles has shown that the MGMT methylation rate in normal lung tissue, NSCLC tissue, plasma, and the bronchial lavage fluid were 16%, 38%, 23%, and 39%, respectively. The results revealed a much higher odds ratio in cancer tissue than that in normal lung tissue and plasma^[30]^ A study^[31]^ on the expression of MGMT promoter methylation in BMs from solid tumors showed that the incidence rate of promoter methylation of lung cancer was the highest (46.5%).

Results also suggest the safety and efficacy of TMZ when used to treat the BMs from NSCLC. Moreover, TMZ prolonged the PFS of intracranial lesions and the OS of the patients. In conclusion, TMZ can be used as a reasonable therapy for the patients suffering from BMs due to NSCLC, especially in patients having no actionable oncogenic driver or resistance to EGFR-TKIs/ALK-TKIs. For patients with BMs, the MGMT promoter methylation status test should also be performed at the same time as molecular testing, to identify patients who would benefit from temozolomide treatment. Further clinical trials are required for identifying the type of BMs from NSCLC much suited for TMZ treatment.

## Author contributions

**Conceptualization:** Mingfang Xu.

**Data curation:** Yi Yang.

**Formal analysis:** Yi Yang.

**Investigation:** Yu Pu.

**Methodology:** Yu Pu, Mingfang Xu.

**Project administration:** Yu Pu, Mingfang Xu.

**Resources:** Yu Pu.

**Software:** Nan Dai.

**Supervision:** Nan Dai.

**Validation:** Nan Dai.

**Visualization:** Mingfang Xu.

**Writing – original draft:** Mingfang Xu.

**Writing – review & editing:** Dong Wang, Mingfang Xu.
